# Asymmetric expansions of FT and TFL1 lineages characterize differential evolution of the EuPEBP family in the major angiosperm lineages

**DOI:** 10.1186/s12915-021-01128-8

**Published:** 2021-08-31

**Authors:** Tom Bennett, Laura E. Dixon

**Affiliations:** School of Biology, University of Leeds, Leeds, LS2 9JT UK

**Keywords:** FLOWERING LOCUS T, Phosphatidylethanolamine-binding proteins, Evolution, Flowering plants

## Abstract

**Background:**

In flowering plants, precise timing of the floral transition is crucial to maximize chances of reproductive success, and as such, this process has been intensively studied. FLOWERING LOCUS T (FT) and TERMINAL FLOWER1 (TFL1) have been identified as closely related eukaryotic phosphatidylethanolamine-binding proteins (‘EuPEBPs’) that integrate multiple environmental stimuli, and act antagonistically to determine the optimal timing of the floral transition. Extensive research has demonstrated that FT acts similar to hormonal signals, being transported in the phloem from its primary site of expression in leaves to its primary site of action in the shoot meristem; TFL1 also appears to act as a mobile signal. Recent work implicates FT, TFL1, and the other members of the EuPEBP family, in the control of other important processes, suggesting that the EuPEBP family may be key general regulators of developmental transitions in flowering plants. In eudicots, there are a small number of EuPEBP proteins, but in monocots, and particularly grasses, there has been a large, but uncharacterized expansion of EuPEBP copy number, with unknown consequences for the EuPEBP function.

**Results:**

To systematically characterize the evolution of EuPEBP proteins in flowering plants, and in land plants more generally, we performed a high-resolution phylogenetic analysis of 701 PEBP sequences from 208 species. We refine previous models of EuPEBP evolution in early land plants, demonstrating the algal origin of the family, and pin-pointing the origin of the FT/TFL1 clade at the base of monilophytes. We demonstrate how a core set of genes (MFT1, MFT2, FT, and TCB) at the base of flowering plants has undergone differential evolution in the major angiosperm lineages. This includes the radical expansion of the FT family in monocots into 5 core lineages, further re-duplicated in the grass family to 12 conserved clades.

**Conclusions:**

We show that many grass FT proteins are strongly divergent from other FTs and are likely neo-functional regulators of development. Our analysis shows that monocots and eudicots have strongly divergent patterns of EuPEBP evolution.

**Supplementary Information:**

The online version contains supplementary material available at 10.1186/s12915-021-01128-8.

## Background

In flowering plants (angiosperms), precise timing of the floral transition is crucial to maximize chances of reproductive success. During the domestication and subsequent improvement of crop species, there has been intensive selection for alteration in this timing to enable higher yield potential in different climatic regions. As such, floral transition and flower emergence are very intensively studied aspects of plant development. At least seven different environmental and developmental stimuli feed into the decision to flower, including day length, temperature, age and vernalization [1]. In the model plant *Arabidopsis thaliana* (‘Arabidopsis’), these signaling pathways regulate the expression of a small number of ‘floral integrators’ including *SUPRESSOR OF OVEREXPRESSION OF CONSTANS 1* (*SOC1*) and *FLOWERING LOCUS T* (*FT*). FT belongs to a family of highly conserved eukaryotic phosphatidylethanolamine-binding proteins (‘EuPEBPs’) in plants (Interpro classification IPR035810). These are quite distinct from ‘bacterial’ PEBPs of the YbhB/YbcL class (Interpro classification IPR005247), which are also found in plants, and which will not be discussed further here. FT and its orthologues have been identified as key regulators of flowering time in many angiosperm species [2]. Extensive research has demonstrated that FT acts similar to a hormone, being transported in the phloem from its primary site of expression in leaves to its primary site of action in the shoot meristem [3]. As such, it has been identified as a key contributor to the mobile ‘florigen’ signal, identified in the 1930s by grafting studies [4]. The *FT* gene acts as a hub for environmental signal integration in leaves, while the FT protein acts in shoot meristems to induce the change from the vegetative to the reproductive developmental program in newly formed tissues.

In Arabidopsis, there are five other EuPEBPs; *TWIN SISTER OF FT* (*TSF*), a recent and partially redundant duplicate of *FT* within the Brassicaceae [5]; *TERMINAL FLOWER 1* (*TFL1*), *ARABIDOPSIS THALIANA CENTRORADIALIS* (*ATC*), *BROTHER OF FT AND TFL1* (*BFT*) and *MOTHER OF FT AND TFL1* (*MFT*) [6, 7]. *MFT* has been characterized as the ancestral form, since EuPEBPs in basally diverging land plants are more structurally similar to MFT than FT/TFL1. MFT appears to play a conserved role in regulating seed dormancy across flowering plants [8, 9]. In Arabidopsis, TFL1 fulfils the opposite role to FT, acting to delay the floral transition [10, 11]; *tfl1* mutants flower immediately after germination. In *Antirrhinum majus*, CENTRORADIALIS (CEN) has a very similar function to TFL1. It was initially believed these were orthologues [11] but further research indicated that TFL1 and CEN belong to separate, but very closely related EuPEBP clades [12]. BFT is also closely related to TFL1 and CEN, and all three proteins act in a similar and partly redundant manner to repress flowering, albeit in response to different stimuli [12–14].

In Arabidopsis, once FT is transported to the shoot meristems, it forms part of a ‘floral activating complex’ by binding with the bZIP transcription factor FLOWERING LOCUS D (FD) and 14-3-3 proteins, and this complex directly regulates the expression of genes involved in reproductive development [15]. TFL1, CEN and BFT act to repress the floral transition by directly competing with FT for the binding of FD and 14-3-3 proteins [13, 14] and form a ‘floral repressing complex’ [16]. This mechanism has been shown to regulate flowering time across angiosperms, indicating its conserved nature [17–20]. At the sequence level, FT and TFL1/CEN/BFT proteins are 98% similar and differences in a few key amino acids have been suggested to account for the functional divergence of these genes. With reference to the Arabidopsis FT sequence, Y134 and W138 in the P-loop region, Y85, Q140 and the conserved ‘LYN’ triad (L150, Y151 and N152) are all strongly indicative of a floral activator function [21–23]. Other key amino acids include those required for the 14-3-3 interaction; R62, T66, P94, F101 and R130, with a further five being identified as facilitating this interaction (positions 60, 61, 64, 96 and 97) [17, 18].

In several species, duplicated paralogues of FT have been converted to alternative functions. For instance, in potato, the FT paralogue SP6A acts independently of the florigen-like FT protein SP3D to promote tuber formation, rather than flowering [24, 25]. A similar situation is found in onion, where one FT paralogue promotes flowering, one promotes bulb formation, and another represses bulb formation [26, 27]. There has also been an expansion of the FT family in grasses [28], and these duplicated paralogues play many different roles, including determining floral meristem transition stages [29, 30] and the integration of specific environmental signals [31]. Thus, while EuPEBPs are crucial in the regulation of flowering time, they also have a larger range of regulatory functions in developmental transitions beyond this, e.g. [25, 26, 31–35].

Their key role in flowering time regulation, as well as the distinction in function in the EuPEBP family despite high sequence similarity, has led to extensive interest in the evolution of the EuPEBP family. Early studies indicated the tripartite division of the EuPEBP family in angiosperms into the FT, TFL1/CEN/BFT (TCB) and MFT clade, with FT and TCB more closely related to each other than MFT [6, 36–38]. Hedman et al. [39] subsequently showed that EuPEBPs are likely present across land plants, but that only MFT-like proteins were present in basally diverging lineages. The evolution of the FT/TFL1 lineage was thus posited to occur in seed plants [39], an idea supported by the identification of a clade of EuPEBPs in gymnosperms that appeared to be equally related to FT and TFL1 [40, 41]. This led to the idea that duplication and functional divergence of FT and TFL1 only occurred in angiosperms; functional analysis of gymnosperm FT/TFL1 in Arabidopsis suggested they acted as floral repressors similar to TFL1 [40, 41]. However, with the increasing availability of sequence data from gymnosperms, Liu et al [42] subsequently demonstrated that gymnosperms in fact possess distinct FT and TFL1 proteins, showing that MFT-FT/TFL1 and FT-TFL1 duplications both predate the origin of seed plants.

The changing picture of EuPEBP evolution in land plants over the last decade illustrates the difficulties in understanding evolutionary history when sampling from a limited number of species with sequenced genomes. There remain a large number of unanswered questions regarding EuPEBP evolution, including the origin of the EuPEBP family, the origin of the FT/TFL1 lineage and the remarkable expansion of the FT family in grasses. In this study, we have taken advantage of a plethora of new genomic and transcriptomic sequence data from across land plants [43–45] to systematically investigate the evolution of the EuPEBP family in land plants as a whole. Using this resource, we have investigated the complex patterns of EuPEBP evolution in angiosperms and how these differ between monocots and eudicots.

## Results

### Canonical EuPEBP proteins are found across the streptophyte lineage

To understand the evolution of the EuPEBP family with greater resolution, we obtained 701 sequences from 208 species, covering all major land plants groups, including charophyte algae (summarized in Additional File 1). We identified unambiguous EuPEBP sequences from the Klebsormidiales, Zygnematales and Coleochaetales, although not the Charales, likely due to the paucity of sequence data for this group. These charophyte sequences show sequence similarity with MFT proteins, consistent with the previously defined MFT-like nature of EuPEBP proteins in early-diverging land plants. Across the 172-amino acid character set that we used for protein analyses (see below and Additional File 2), the charophyte ‘proto-MFT’ proteins share on average 50% identity with Arabidopsis MFT. This compares to the 45.6% and 49.1% identity that AtMFT shares with AtFT and AtTFL1 respectively (Additional File 2). The charophyte sequences are themselves quite diverse in primary protein structure, only having 55.5% identity between them over the same character set. We also identified PEBP proteins in the genomes of chlorophyte algae, but outside the core phosphatidylethanolamine-binding domain, these bear little resemblance to the MFT-like proteins in the charophytes (Additional File 2). Our analysis places the origin for the plant EuPEBP family in the common ancestors of chlorophyte and streptophyte algae and show that the canonical EuPEBP structure found in land plants was already present at the base of the streptophyte lineage.

### Definition of land plant EuPEBP clades

To reconstruct the evolution of the EuPEBP family, we performed phylogenetic analyses on the retrieved sequences using maximum likelihood (ML) approaches at the nucleotide level. These analyses identified a core set of well-resolved clades representing the major land plant lineages (Table 1). In liverworts, mosses, hornworts and lycophytes, we only identified a single clade present in each lineage; all of these proteins resembled angiosperm MFT proteins, as previously described (Table 1) [39]. For monilophytes, gymnosperms and angiosperms, we identified multiple well-resolved clades of proteins; while some of these have previously been described [39, 42], others are identified for the first time here.

**Table 1:** Table summarizing the clades identified in the major streptophyte lineages in this study, and the number of sequences representing the clade in each lineage. Major sub-clades present within subdivisions of the major lineages are also indicated

	Clade	Taxon	Sequences	Major sub-clades
	*prMFT*	Klebsormidiales	1	
	*prMFT*	Charales	0	
	*prMFT*	Coleochaetales	1	
	*prMFT*	Zygnematales	4	
***UrMFT***	*MFT*	Liverworts	2	
	*MFT*	Mosses	4	
	*MFT*	Hornworts	2	
	*MFT*	Lycophytes	14	
***MFT***	*MFT*	Monilophytes	10	
	*MFTA*	Gymnosperms	9	
	*MFTB*	Gymnosperms	47	
	*MFT1*	Angiosperms	56	
	*MFT2*	Angiosperms	12	
***PFT***	*PFT*	Monilophytes	11	
	*TCB*	Gymnosperms	15	
	*TCB*	Angiosperms	165	TCB1, TCB2, BFT, CEN, TFL1
	*FTA*	Gymnosperms	12	
	*FTB*	Gymnosperms	18	
	*FT*	Angiosperms	324	FT1, FT2, FT10, FTX, FTY

### The FT/TFL1 lineage evolved at the base of the euphyllophytes

**Fig. 1:**
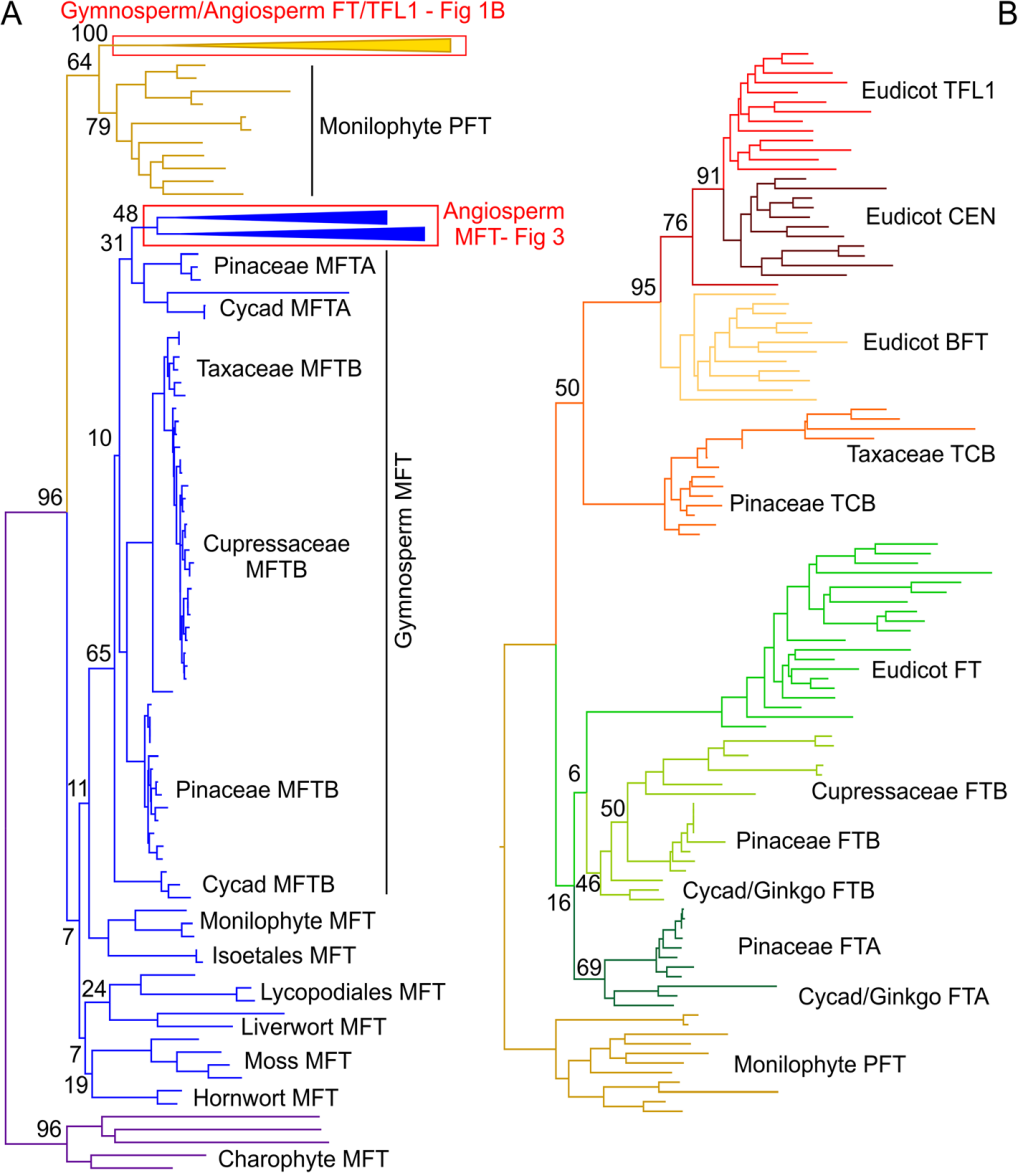
Overall Topology of the EuPEBP family in streptophytes. **A** Nucleotide-level maximum likelihood analysis implemented in PhyMLusing a GTR + G + I model, on the land plant *EuPEBP* family (180 sequences, 519 characters). The tree was rooted with charophyte algal sequences. Phylo/cladogram showing the most likely tree, including bootstrap values at key nodes. Several clades are collapsed, and shown in other figures as indicated. **B** Nucleotide-level maximum likelihood analysis implemented in PhyML using a GTR + G + I model, showing the euphyllophyte *FT/TFL1* clade, which is collapsed in **A**; 111 sequences, 519 characters. The tree was rooted with monilophyte *PFT* sequences. Phylogram showing the most likely tree, including bootstrap values at key nodes

We recovered these core clades irrespective of the phylogenetic approach used, but the relationship between these clades differed dramatically depending on the approach and on the set of sequences and characters used. In particular, the high sequence bias towards the angiosperms (578 sequences), in particular the monocots (350) and especially the Poaceae (263) appeared to make accurate reconstruction of the relationship between the clades highly problematic. Reducing the number of angiosperm sequences resulted in a more consistent topology for the whole family phylogeny. We therefore opted to make separate reconstructions, only bringing in all the angiosperm sequences once we had established an overall topology for the family. We reconstructed the evolution of the whole land plant EuPEBP family with more even sampling across taxonomic groups. After trialing various sets of sequences, a final set of 180 were chosen. These included almost all identified non-angiosperm sequences (123 sequences), plus 57 angiosperm sequences representing basal angiosperms, basal eudicots, euasterids and eurosids. These sequences were trimmed to 519 highly conserved nucleotides (i.e. 173 amino acids) based on their amino acid alignment (Additional File 3). PhyML was then used to reconstruct the most likely phylogenetic topology, using a GTR + G + I model selected in Jmodeltest 2. When rooted with the charophyte sequences, the resulting topology is congruent with previous analyses [39–42] and shows the classic tripartite division of the family into MFT, FT and TCB (TFL1/CEN/BFT) lineages within seed plants (Fig. 1A).

**Fig. 2:**
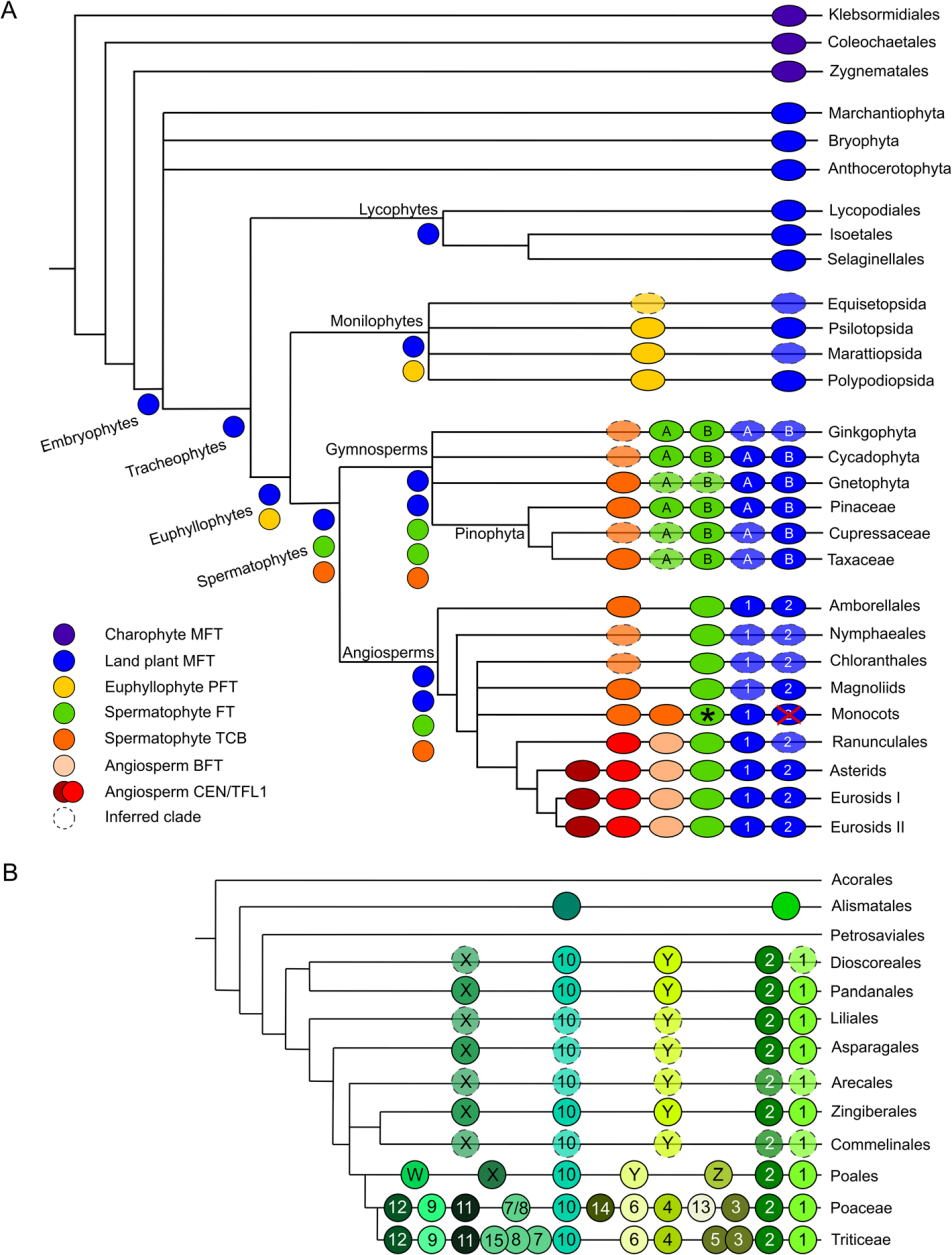
Reconstructed evolution of the EuPEBP family in streptophytes. **A** Schematic depicting the complement of EuPEBP proteins in major land plant and charophyte algae groups, and their inferred evolutionary origin. Each branch indicates a major streptophyte lineage; lycophytes, monilophytes and gymnosperms are further sub-divided into relevant orders/families/etc. The circles on each branch indicate the core complement of proteins in that group or sub-group. Clades which are inferred by parsimony are denoted with a translucent circle, and clades believed to have been lost are shown with a red cross. Letters and numbers in the circles indicate clade names. Circles at internal branching points represent the minimum inferred EuPEBP protein complement in the last common ancestor of each major land plant group. Asterisk: Refer to **B**. **B** Schematic depicting the complement of FT proteins in monocot orders, and within the Poales the family Poaceae and tribe Triticeae. Each branch indicates a monocot order (etc.). The circles on each branch indicate the core complement of proteins in that group or sub-group. Clades which are inferred by parsimony are denoted with a translucent circle. Letters and numbers in the circles indicate clade names

As we have previously observed for reconstructions of land plant gene families [46–48], there is generally low bootstrap support along the backbone of the phylogenetic tree (Fig. 1A). There are also predictable issues at the base of the tree, with the separation of sequences from the lycophyte orders (selaginellales, isoetales and lycopodiales) and the attraction of some of these sequences to the base of the tree. Even if reconstructed using only sequences from liverworts, mosses, hornworts, lycophytes and monilophytes, these same issues occur (Additional File 4); the divergence in sequences between these groups is too large for accurate reconstruction. However, given that the liverworts, mosses, hornworts and lycophytes only have one identifiable clade of EuPEBP proteins, there is little controversy about the evolution of the family up to the base of the euphyllophytes (Fig. 2).

Unlike these aforementioned groups, monilophytes have two well-resolved clades of EuPEBP proteins in our reconstructions; these do not group with each other, but are separated into distinct branches of the tree (Fig. 1A). One monilophyte clade groups with MFT sequences from gymnosperms and angiosperms, while the other groups with the FT and TFL1 sequences from gymnosperms and angiosperms (Fig. 1A, B). This existence of this ‘PROTO FT & TFL’ (PFT) clade of proteins in monilophytes strongly suggests that (1) the duplication that led to separate MFT and FT/TFL1 lineages occurred at the base of the euphyllophytes, and (2) the duplication that led to separate FT and TFL1 lineages occurred after the divergence of monilophytes and seed plants (Fig. 2). Thus, our data clearly define when the first two key duplications in the evolution of the EuPEBP family occur. Consistent with their phylogenetic position, the PFT proteins have a mix of characters, some in common with MFT (especially 84 W) and some in common with FT/TFL; the proteins are neither FT-like or TFL1-like (Additional File 3).

### FT and MFT are ancestrally duplicated in gymnosperms

We identified five major clades of EuPEBP proteins in gymnosperms; a single TCB clade and two clades within the FT lineage (FTA, FTB), consistent with the analysis of [42] (Fig. 1A, B; Table 1). We also identified two MFT lineages in gymnosperms (MFTA, MFTB), which were not defined in Liu et al, but which are apparent in that study [42]. We identified each of these five clades in at least three of the main gymnosperm divisions (i.e. Ginkgophytes, Cycadophytes, Gnetophytes, Pinophytes), suggesting they represent a core complement of proteins present in the last common ancestor of extant gymnosperms. This is strongly supported by our phylogenetic reconstruction, which shows the MFTA/MFTB and FTA/FTB duplications are long-standing and occur at the base of the gymnosperm group (Fig. 1A, B).

### MFT is independently duplicated in angiosperms

**Fig. 3:**
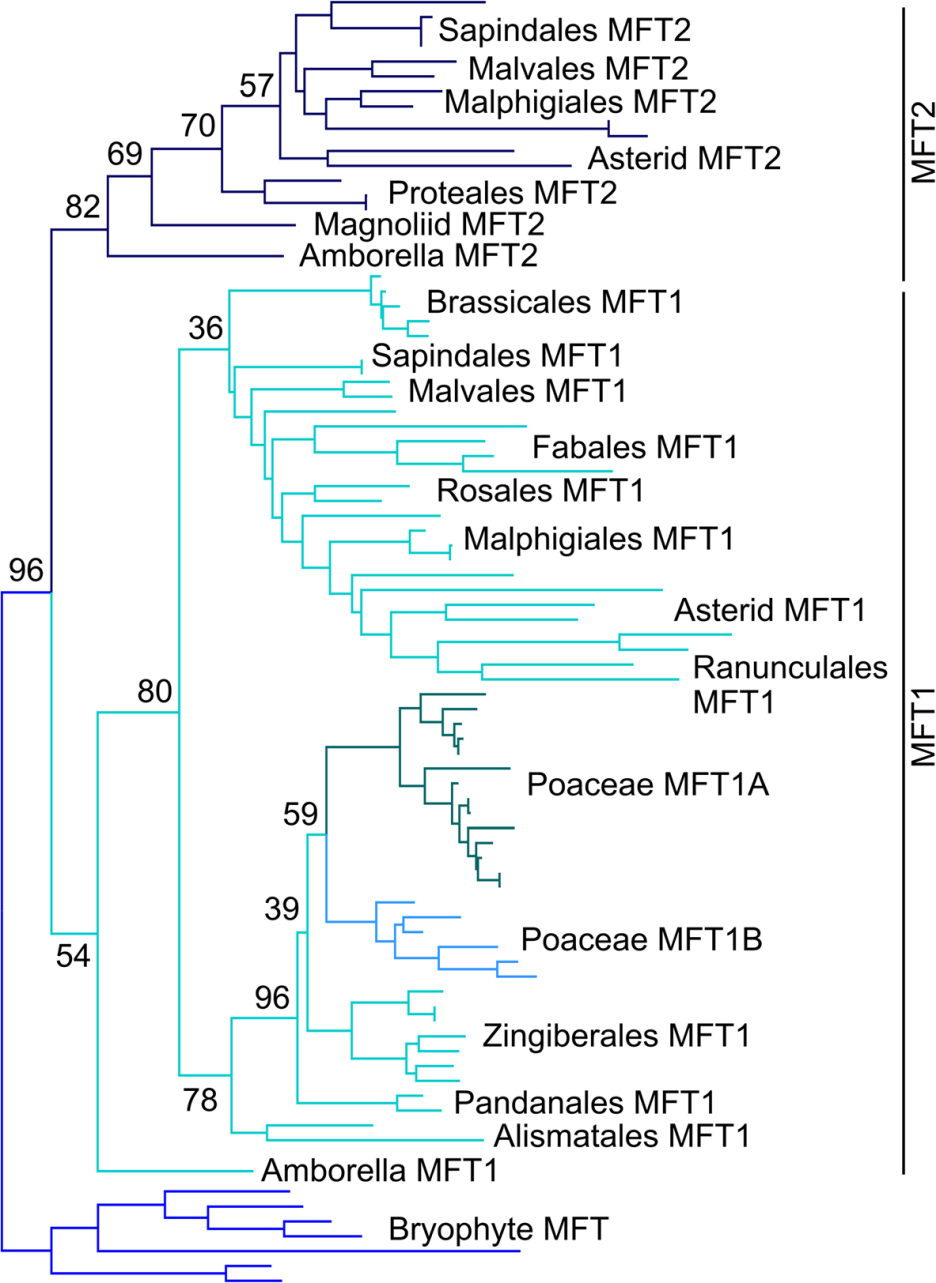
Evolution of the MFT lineage in angiosperms. Nucleotide-level maximum likelihood analysis implemented in PhyMLusing a K80 + G + I model, on the angiosperm MFT clade (77 sequences, 513 characters). The tree was rooted with bryophyte MFT sequences. Phylogram showing the most likely tree, including bootstrap values at key nodes

Consistent with analysis of [39], we also identified two distinct clades of MFT proteins (MFT1/MFT2) in angiosperms (Table 1). Our phylogenetic reconstruction shows that this represents a duplication in the ancestor of the angiosperms that is independent of the equivalent duplication in gymnosperms (Fig. 1A, Fig. 2). To understand this more clearly, we undertook a more focused reconstruction of the MFT family in angiosperms, this time including all angiosperm sequences. We used a slightly reduced 513 nucleotide character compared to our main reconstruction, with 77 sequences from across angiosperms, and 7 bryophyte MFTs as an outgroup (Additional File 5). PhyML was then used to reconstruct the most likely phylogenetic topology, using a K80 + G + I model selected in Jmodeltest 2. This reconstruction supports the conclusion of an ancestral duplication in angiosperms, with MFT1 and MFT2 proteins both present in the basal angiosperm *Amborella trichopoda*, as well as the magnoliids, basal eudicots and core eudicots including both asterids and rosids (Fig. 3). However, MFT2 appears to have been lost relatively frequently from genomes compared to MFT1 and is not present in some key angiosperm groups. Although monocots have lost MFT2, a later duplication within the MFT1 clade in the Poaceae means two MFT clades (MFT1A/MFT1B) are present across the grass family (Fig. 3).

### Independent expansions of the TCB lineage in monocots and eudicots

**Fig. 4:**
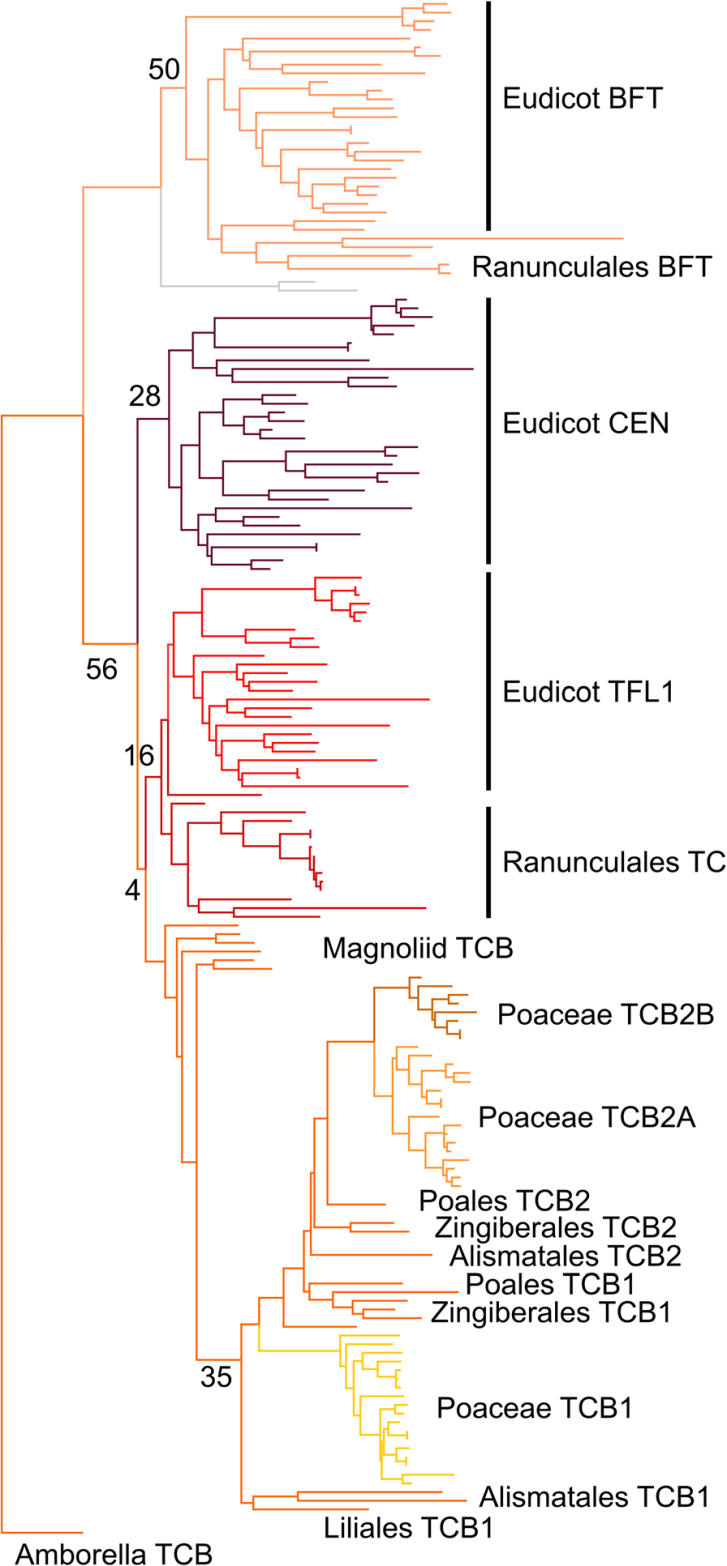
Evolution of the TCB lineage in angiosperms. Nucleotide-level maximum likelihood analysis implemented in PhyMLusing a TVM + G + I model, on the angiosperm TCB clade (165 sequences, 507 characters). The tree was rooted with *Amborella trichopoda TCB*. Phylogram showing the most likely tree, including bootstrap values at key nodes

Core eudicot model species such as Arabidopsis have been found to have three distinct proteins representing the TCB clade of the EuPEBP family. However, the evolutionary events that led to this tripartite structure are unclear. We therefore reconstructed the evolution of the TCB lineage in angiosperms, this time including all angiosperm sequences. We used a slightly reduced 507 nucleotide character set compared to our main reconstruction, with 169 sequences from across angiosperms (Additional File 6). PhyML was then used to reconstruct the most likely phylogenetic topology, using a TVM + G + I model selected in Jmodeltest 2. Because of the high level of protein identity between all proteins in this clade, reconstructing the evolution of the family was difficult. In a trial amino acid-based reconstruction, there were extensive polytomies, and even using nucleotide data, the number of informative characters was relatively small. The resulting phylogenetic topology suffers from some issues, with non-core-eudicot sequences tending to group with TFL1, even though this is improbable from an evolutionary perspective (Fig. 4). However, based on the groups of sequences present in each angiosperm, the evolution of the family can be satisfactorily resolved. Gymnosperms only have a single clade of TCB proteins, and consistent with this *Amborella trichopoda* only has a single TCB protein, suggesting this was the ancestral state in angiosperms too. Conversely, as expected, core eudicot TCB proteins grouped into three distinct TFL1, CEN and BFT clades, with TFL1 and CEN more closely related to each other than to BFT (Fig. 4). In the basal eudicot order of the Ranunculales, we identified two distinct clades of proteins, one of which unambiguously grouped with the core eudicot BFT sequences (Fig. 4). The other clade grouped with the TFL1 clade, but more likely is equally related to TFL1 and CEN, given the absence of any CEN-like sequences in the Ranunculales. This suggests that the duplication that created TFL1 and CEN clades occurred after the divergence of the Ranunculales and the core eudicots (Fig. 2). We identified a large number of TCB sequences from across monocots, which unambiguously grouped into a single clade. This therefore suggests that the duplication that created the TFL/CEN and BFT clades occurred after the divergence of monocots and eudicots (Fig. 2). Consistent with this, we also identified a single group of TCB proteins from magnoliids (arranged as a grade in Fig. 4). Within the monocot clade, we found clear evidence for an early duplication in the TCB family in monocots (Fig. 2, Fig. 4). For the orders Alismatales, Zingiberales and Poales, there were two distinct clades of TCB proteins (TCB1 and TCB2) present in the phylogeny. The TCB2 proteins form a coherent clade in Fig. 4, while the TCB1 proteins are arranged as a grade relative to this. Since the Alismatales are a basal monocot order, the duplication that led to the TCB1 and TCB2 clades must have occurred very early in monocot evolution. In the Poaceae, we identified a further duplication in the TCB2 clade (not present in the broader Poales), meaning that all grasses have 3 TCB proteins, equivalently to, but independently of, the core eudicots.

### Radiation of the FT lineage in monocots

**Fig. 5:**
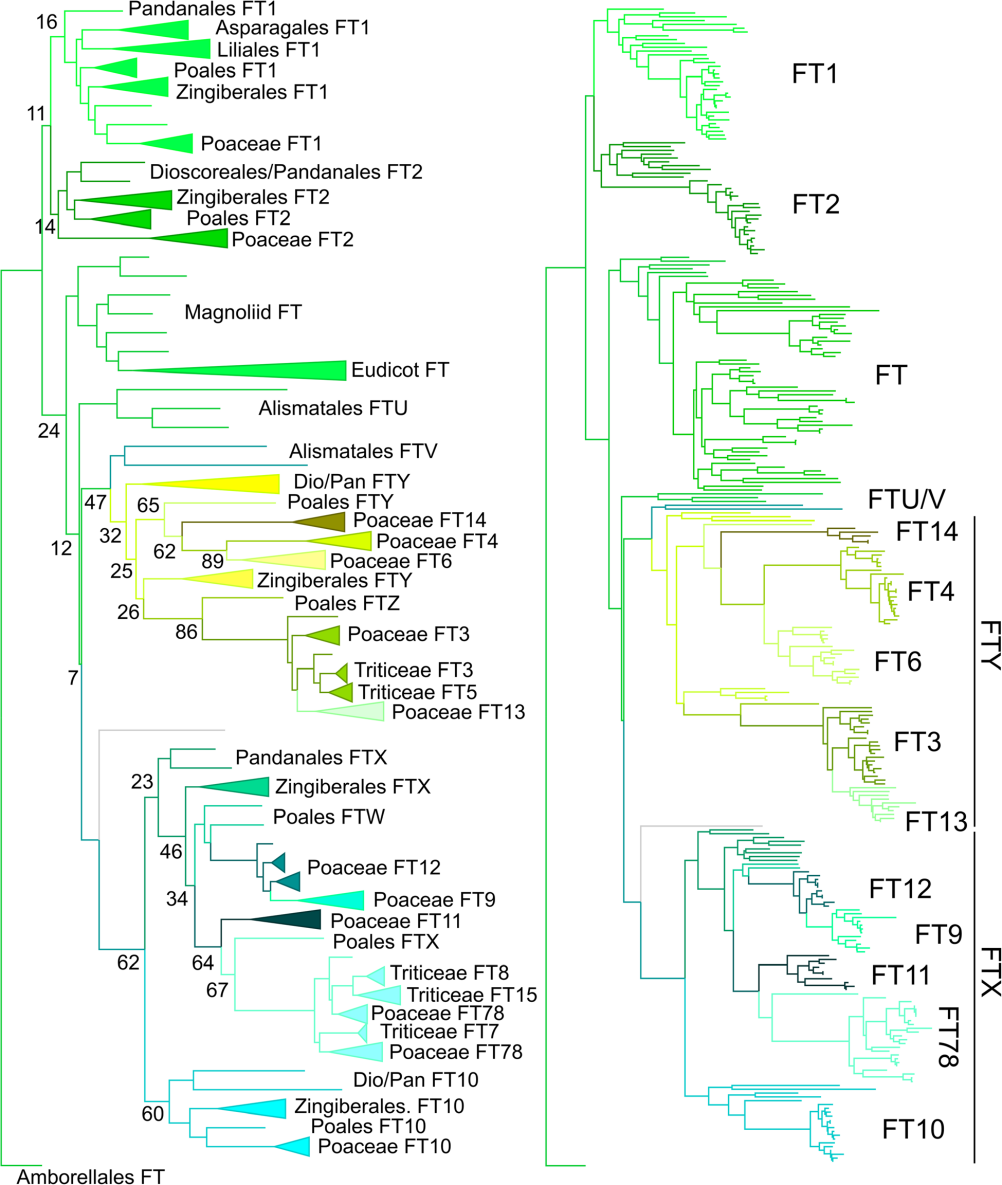
Evolution of the FT lineage in angiosperms. Nucleotide-level maximum likelihood analysis implemented in PhyMLusing a TVM + G + I model, on the angiosperm FT clade (304 sequences, 507 characters). The tree was rooted with *Amborella trichopoda FT*. **A** Cladogram showing the most likely tree, including bootstrap values at key nodes. Major clades are collapsed and labelled. **B** Phylogram showing the most likely tree, showing the interrelationship of clades in the Poaceae

Finally, we attempted to understand the evolution of the FT lineage. In eudicots, FT genes are generally present either as a single copy, or as a pair of recent paralogs (e.g. FT/TSF in Arabidopsis), but it is clear that large numbers of FT paralogs are present in the genomes of the Poaceae [28]. To understand the events that led to this dramatic difference in FT composition, we reconstructed the evolution of the FT lineage in angiosperms, this time including all angiosperm sequences. We used a slightly reduced 513 nucleotide character compared to our main reconstruction, with 304 sequences from across angiosperms (Additional File 7). PhyML was then used to reconstruct the most likely phylogenetic topology, using a TVM + G + I model selected by Jmodeltest 2. Although more complex than the TCB lineage, the higher levels of protein divergence between clades in the FT lineage made it easier to reconstruct its evolution. As with TCB, *Amborella trichopoda* only has a single FT protein, suggesting this was the ancestral state in angiosperms. We found that all magnoliid, basal eudicot and core eudicot FT proteins formed a single clade, with no evidence of any major duplications in the clade (though some individual families may have duplications, as with the FT-TSF duplication in Brassicaceae) (Fig. 5). Thus, through most of angiosperm evolution, FT has been maintained as a single copy gene (Fig. 2). In contrast with this, we detected a remarkable 12 distinct clades of FT proteins in the Poaceae (FT1, 2, 3, 4, 6, 7/8, 9, 10, 11, 12, 13 and 14) (Fig. 5). While [28] previously identified 12 FT genes in *Hordeum vulgare* (barley) and *Triticum aestivum* (bread wheat), our 12 clades are not completely co-equal to these. For instance, the FT13 and FT14 clades that we define for the first time here appear to have been lost from the Triticeae, so do not appear in previous analyses of wheat and barley. Conversely, the gene pairs FT3/FT5 and FT7/FT8 present in Triticeae represent single genes (FT3, FT7/8) in other tribes of the Poaceae (Fig. 2). Indeed, we show that FT7/8 has become a gene triplet in the Triticeae, with a third co-ortholog of FT7 and FT8 (FT15) present in both wheat and barley (Fig. 2, Fig. 5).

Given the striking expansion in FT gene number in monocots, we asked if this represented a sustained increase in FT gene number during monocot evolution or a sudden burst. A sudden burst could be potentially linked to major selection events, although we do not test that here. In the basal monocot order of the Alismatales, there are two distinct clades present, which we have named FTU and FTV (Fig. 2, Fig. 5), showing that the first FT duplication occurred at the base of the monocots. In the mid-diverging order of the Pandanales, we identified 5 distinct groups of genes (FT1, FT2, FT10, FTX and FTY) (Fig. 5). We identified the same 5 groups collectively between the other mid-diverging orders of the Dioscoreales (FT2, FT10, FTY), Liliales (FT1, FT2) and Asparagales (FT1, FT2, FTX). The same five groups were also present in the commelinid crown group order of the Zingiberales (Fig. 5). Thus, after the divergence of the Alismatales and other monocots, there was a rapid expansion from 2 to 5 FT genes. This was complete before the divergence of the Dioscoreales/Pandanales from other lilioid monocots (Fig. 2) and was maintained throughout subsequent monocot evolution into the commelinid crown group. In contrast to the Zingiberales, we identified 7 distinct clades within the Poales, a sister clade within the commelinid crown group. This occurred by duplication of FTX and FTY into four paralogs, FTW, FTX, FTY and FTZ, which are present through the Poales; FT1, FT2 and FT10 are also present, unduplicated (Fig. 2, Fig. 5). Within the Poaceae, these 7 clades then became amplified into the 12 core FT clades described previously. FTW was duplicated to give rise to the FT9 and FT12 clades, while FTX gave rise to the FT7/8 (further duplicated in the Triticeae) and FT11 clades. Similarly, FTY was triplicated to give rise to FT4, FT6 and FT14, while FTZ was duplicated to form FT3 (further duplicated in the Triticeae) and FT13.

### Conservation of distinctive features in different EuPEBP lineages

**Fig. 6:**
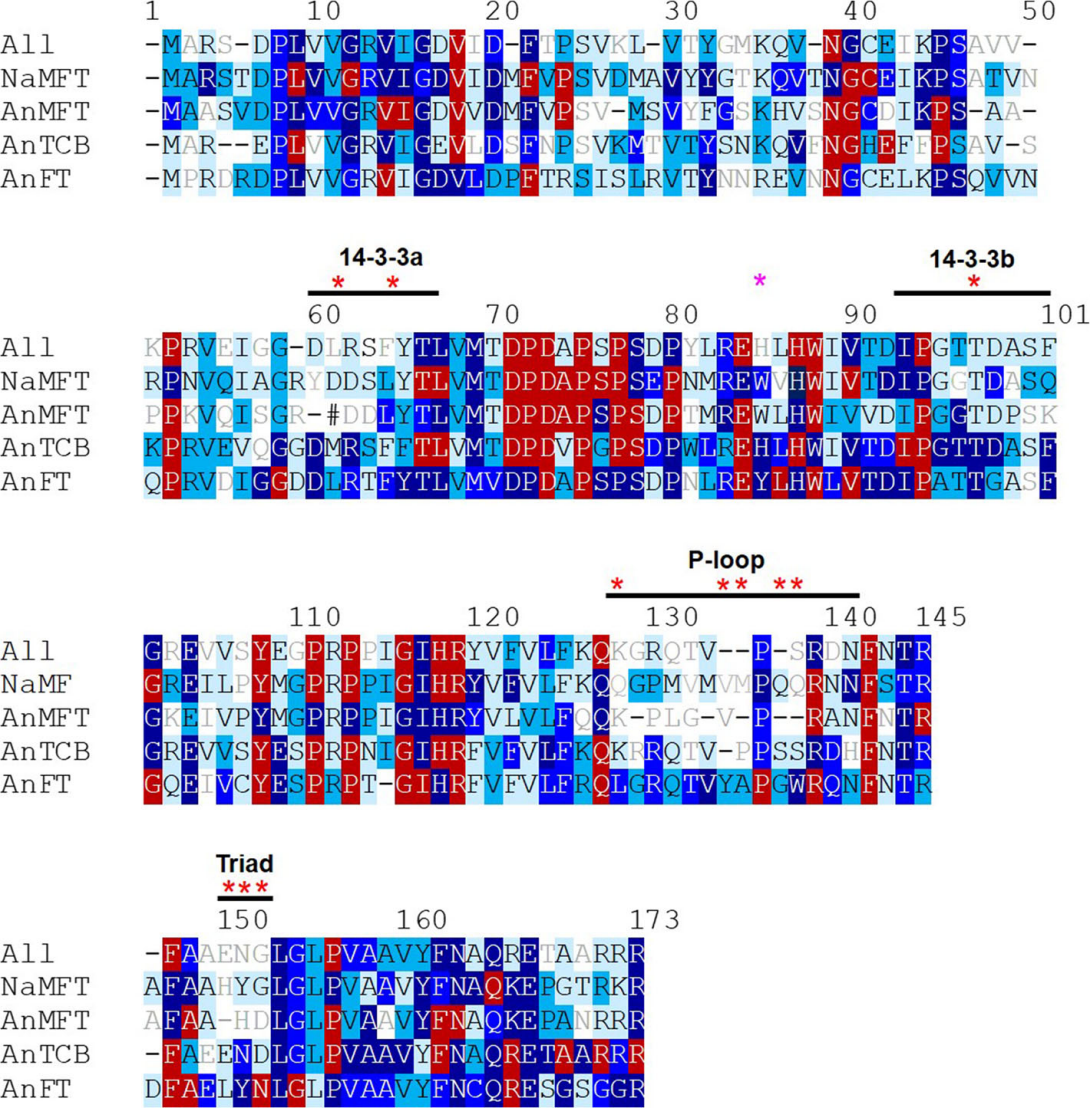
Conserved and unique characteristics of EuPEBP protein families. Alignment illustrating conservation of primary protein structure in EuPEBP proteins. In total, 172 core residues (numbered relative to Arabidopsis thaliana FT, for the sake of consistency with previous literature) are shown in the alignment, for the whole family (All), for non-angiosperm MFT (NaMFT), and for angiosperm MFT, TCB and FT proteins (AnMFT, AnTCB, AnFT respectively). Residues where the same amino acid is present in >30% are shown by the corresponding amino acid letter. Where there is no consensus at all, – is shown, where a residue is absent, #. Where there is >30% consensus, but <50% ('weak consensus') the amino acid is shown in grey without shading. Residues with >50% consensus are shaded: light blue 75% < 50% ('consensus'); mid-blue 90%<75% (‘conserved’), royal blue 95%<90% (‘highly conserved’), dark blue 99%<95% (‘very highly conserved’), dark red >99% (‘invariant’). Structural features are annotated above the alignment, including the 14-3-3 interaction surface (in two parts, a and b), the P-loop motif and the ‘LYN triad’. Key amino acids previously identified as conferring specificity are indicated by *

The dataset we compiled presented us with the opportunity to define key residues in all types of EuPEBP protein, by looking at conservation in amino acid usage across the whole family. To perform these analyses, we used a 172-amino acid character set, essentially identical to those used for all phylogenetic reconstruction. These 172 residues are those which are present in essentially all members of the land plant EuPEBP family; individual proteins might have additional residues at the N- or C-terminus, or within the protein, but these represent the core, conserved structural elements. For each of these 172 residues, we then assessed the relative proportion of each amino acid present. If a single type of amino acid was present at a position in > 30% sequences, we deemed it a ‘weak consensus’ (Fig. 6, grey text); a ‘consensus’ if present in > 50% sequences (Fig. 6, light blue shading), and ‘conserved’, ‘highly conserved’, ‘very highly conserved’ and ‘invariant’ if present in > 75%, > 90%, > 95% and > 99% of sequences respectively (Fig. 6, mid-blue, royal blue, dark blue and red shading respectively).

We first performed this analysis across the whole family (but leaving aside the monocot FTs in the interim). Across the 420 sequences included in this analysis, 22 residues were completely invariant, another 77 were conserved, highly conserved or very highly conserved, and only 10 residues had no consensus at all (Fig. 6). This emphasizes the very strong conservation of primary protein structure within the family as a whole. We then repeated this analysis for each of the angiosperm clades (AnMFT, AnTCB, AnFT), as well as 87 non-angiosperm MFT proteins as a paraphyletic group (NaMFT). Interestingly, the AnMFT clade shows no more conservation than the family as whole, with 26 invariant resides, 69 conserved, highly conserved or very highly conserved residues, and 10 residues with no consensus (Fig. 6). In contrast, the TCB clade shows higher levels of conservation with 34 invariant residues, 78 conserved, highly conserved or very highly conserved residues and only 5 residues with no consensus, while the FT clade displays exceptional conservation with 28 invariant and 97 conserved residues, and only a single residue lacking consensus (Fig. 6).

This analysis also allowed us to exhaustively define the conserved unique features of each angiosperm clade (Additional File 8). Interestingly, the AnMFT clade has relatively few (8) unique residues not found in other groups, which is substantially less than the 22 unique residues found in the paraphyletic NaMFT group. AnTCB has a moderate number of unique residues (26), while AnFT has 40 unique residues not found in the other groups (Fig. 6, Additional File 8). This suggests that FT has undergone more selection for new functionality compared to TCB, consistent with previous suggestions about the ancestral functions of EuPEBP proteins [40, 41]. It is notable that, in the family as a whole, the conserved residues in the proteins are not evenly distributed, but cluster into distinct highly conserved motifs separated by non-conserved regions. Intriguingly, these non-conserved regions include the previously defined 14-3-3 interaction surface (59–65 and 93–100) and the P-loop (127–140) motif [17, 18, 21]. There are important sequence differences between AnTCB and AnFT proteins in these motifs that contribute to the relative lack of conservation (Supplementary Figure 3), but the main reason for this non-conservation at a whole family level appears to be the lack of conservation in the 14-3-3 interaction and P-loop domains in AnMFT proteins (Fig. 6). In particular, one of the key 14-3-3 residues (60) is completely absent from AnMFT proteins, and none of the 5 key P-loop residues (127, 133, 134, 136, 137) are conserved in AnMFT. Furthermore, the ‘LYN triad’ (149-151) is also non-conserved in AnMFTs. Intriguingly, despite representing a much more diverse group of organisms across the full spread of land plant evolution, the NaMFT proteins showed much clearer conservation in the 14-3-3, P-loop and LYN motifs. Thus, angiosperm MFT proteins appear to have lost their 14-3-3, P-loop, LYN motifs after their divergence from other MFT proteins, possibly reflecting the sub-functionalization that has occurred across the EuPEBP family in angiosperms.

### Expansion of FT in the monocot lineage

**Fig. 7:**
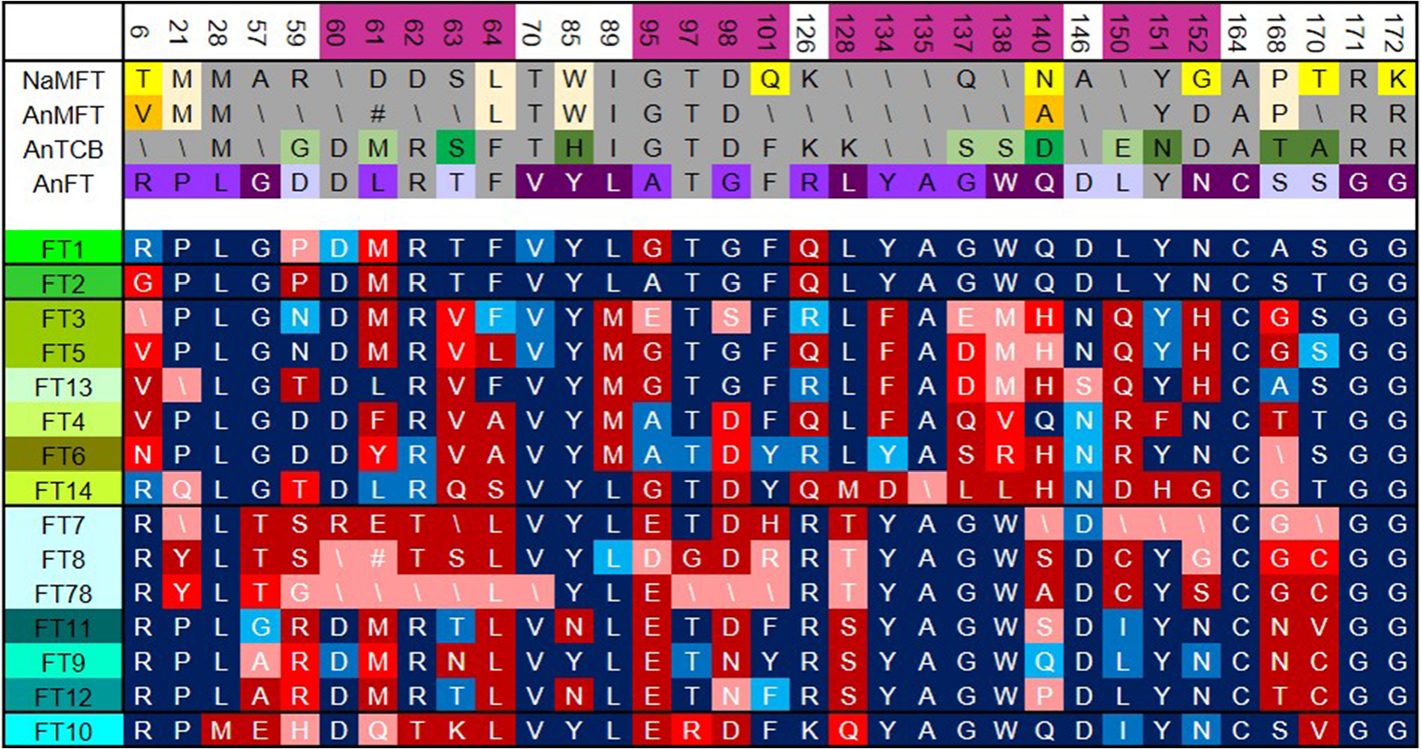
Unique features of grass FT proteins. Diagram showing changes in otherwise conserved residues in FT protein clades from the Poaceae. A total of 33 residues are shown along the top, these are either residues where Angisoperm FT proteins (without the monocots)(AnFT) have a unique amino acid relative to other EuPEBP proteins, or otherwise conserved amino acids in important motifs (shaded pink), 14-3-3a and b (60-64 and 95-101), P-loop (128-140) and LYN triad (150-152). The consensus sequence within non-angiosperm MFT (NaMFT), angiosperm MFT (AnMFT), angiosperm TCB (AnTCB) and AnFT at each residue is shown in rows two to five. Amino acids characteristic for NaMFT are shaded yellow, those for AnMFT are shaded orange, those for TCB are shaded green, and those for AnFT are shaded purple. Residues shared across all MFT proteins are shaded beige. The darker the shade, the higher the degree of conservation within the clade. Residues that are not characteristic are shaded grey; if there is no consensus, a backslash (\) is shown. The following 15 rows indicate the consensus amino acids present at the same residues in the different FT clades in the Poaceae. Blue shading indicates that the consensus matches the consensus for EuFT. Dark blue = highly conserved in clade (> 90%), mid-blue = conserved (> 75%), light blue = consensus (> 50%). Red shading indicates that the consensus does not match the consensus for EuFT. Dark red = highly conserved in clade (> 90%), mid red = conserved (> 75%), pink = consensus (> 50%), or where backslash is shown, no consensus (but therefore still distinct from EuFT), and where # is shown, residue is completely absent

The expansion of FT paralogues in monocots, and especially in grasses, raises important questions as to the function of these additional proteins, and what selective pressures have maintained—and increased—FT copy number in these genomes. Having defined the key features of angiosperm MFT, TCB and FT proteins, we were able to assess whether there was evidence the grass FT proteins are sub- or neo-functionalized. For each residue at which FT has a unique conserved amino acid (i.e. those in Supplementary Figure 3), and for several other key residues in the 14-3-3 interaction, P-loop and LYN motifs, we assessed whether grass FT proteins had FT-like amino acids, or novel amino acid usage (Fig. 7). If there was no difference between eudicot FT (EuFT) and any of the grass FTs, the residues are omitted from Fig. 7.

The results present an intriguing picture of different changes in different domains between the key 5 monocot clades (FT1, FT2, FT10, FTX, FTY). Consistent with their numbering, FT1 and FT2 are most similar to EuFT, and each protein (when viewed as a clade) only has 4 notable differences in FT-unique residues relative to EuFT (Fig. 7). For FT1, two of these changes (L61M and A95G) are minor; L61M is also found in FT2. R6G in FT2 represents a more significant change, as do D59P and R126Q, which are found in both FT1 and FT2. Other than this, FT1 and FT2 perfectly conserve the structure of AnFT. FT10 has an intermediate similarity relative to AnFT across its length, with the majority of significant changes clustering in and around the 14-3-3 interaction motifs (G57E, D59H, L61Q, R62T, F64L, A95E, T97R, G98D), or in the first P-loop residue (L128Q). These non-conservative changes in amino acid strongly imply that FT10 has changed, or lost its 14-3-3 interaction partner relative to AnFT and FT1/FT2 (Fig. 7).

The FTX clade proteins, which are akin to FT10, share the same features as FT10, including non-conservative changes in residues 59, 60, 61, 63, 64, 95, 98 and 128. However, these proteins also have additional changes relative to EuFT, including in the final P-loop residue (140) and residues 168 and 170 at the very tail of the protein (Fig. 7). These changes are enhanced even further in FT7/8, and the Triticeae sub-clades FT7 and FT8. These proteins also have non-conservative changes in residue 21 (P21Y), 101 and two of the ‘LYN’ triad residues (L150C, N152S). In FT7/8, there is no consensus at all in the protein sequence of the 14-3-3 interaction motif, and in FT7, there is no consensus in the LYN triad (Fig. 7). The well-conserved amino acid changes in FT9/FT11/FT12 suggest that these proteins have merely changed interaction partners, but still retain specificity; the complete ‘decay’ of the 14-3-3 interaction motif in FT7/8 suggests that these proteins do not interact with 14-3-3 proteins.

The FTY clade proteins also have very extensive changes relative to EuFT, but in a different pattern to those seen in FTX proteins. While there are some changes in the 14-3-3 motif residues 61, 63, 64, 95 and 98, only T63V occurs broadly across the clade (Fig. 7). More dramatic are the changes in the P-loop, in which residues 134, 136, 137 and 140 have undergone non-conservative changes (Fig. 7). However, P-loop residue 128, which is changed in FT10 and FTX proteins, remains in its ancestral state in FTY protein; an intriguing distinction. The LYN triad is also non-conservatively changed in FTY, especially residue 150 (Fig. 7). Thus, FTY proteins might have swapped 14-3-3 partners, perhaps less dramatically than FTX proteins, but have certainly undergone radical functional changes in the P-loop and LYN motifs. The exact effect of these changes can only be speculated at here, but these data provide clear avenues for new investigation in the structure-function study of monocot FT proteins.

## Discussion

### The evolution of the EuPEBP lineage in land plants

The results presented here provide novel insights into the evolution of the EuPEBP clades in flowering plants as well as furthering our understanding regarding the trajectory of EuPEBP evolution in land plants. We identify that recognizable full-length MFT-like proteins occur throughout charophyte but not chlorophyte algae. Consistent with previous suggestions, but with much wider sampling, we show that the bryophyte lineages (mosses, liverworts and hornworts) and lycophytes only possess MFT-like proteins (Fig. 1) [39]. However, we show for the first time that ferns possess both proteins in both the MFT and FT/TFL1 lineages, indicating that the duplication that created these lineages occurred at the base of monilophytes (Fig. 2). Thus, an event previously suggested to have occurred at the base of seed plants [40–42] occurred substantially earlier in land plant evolution. Our analysis used the three families of EuPEBP proteins from Arabidopsis as the basis for identification of EuPEBPs in other green lineages. Regardless of which protein was used as a target, the same hits were retrieved from each organism, but it remains possible that more divergent EuPEBP sequences were not recovered by our searches, and as such would not have been included in these analysis.

### Independent radiations in seed plant lineages

Consistent with the analysis of [42], our data show that the tripartite division of the EuPEBP family into MFT, FT and TCB lineages was complete by the base of seed plants. However, our analysis also demonstrates that these three lineages have very different evolutionary histories in different seed plant groups. MFT appears to have undergone independent duplications in both gymnosperms and angiosperms (Fig. 2), but then undergone reduction in many angiosperms orders to a single copy. Intriguingly, it is always MFT2 that has been lost, most notably from the monocots. We have previously observed a similar pattern in the evolution of the PIN auxin efflux carrier and D14/KAI2 α/β hydrolase families, in which a deep duplication in angiosperms leads to a pair of related proteins (PIN5/PIN12 and DLK2/DLK3), one of which is then regularly lost from genomes (PIN12 and DLK3), while the other is stably maintained [46, 47], but there is no obvious explanation for this pattern at present. The TCB lineage was stably maintained as a single copy gene in gymnosperms, but underwent independent duplications within monocots and eudicots. A further duplication in core eudicots gave rise to the conserved TFL1-CEN-BFT set of floral repressors. Conversely, FT underwent a major duplication in gymnosperms, but has fundamentally been maintained as a single copy gene in angiosperms (although there are many examples of local FT duplications in eudicot families), with the startling exception of the monocots.

### The monocot FT expansion

Our most remarkable finding is the dramatic expansion of the FT lineage across the monocot group. At the base of monocots, an initial duplication led to two FT sub-clades (FTU and FTV), before an expansion to five clades in most monocot orders. Due to the still-fragmentary genomic/transcriptomic resources available in basal and medial monocot lineages, we have been unable to precisely pinpoint the timing of the original duplication in the FT lineage, nor the points at which the lineage expanded to five members. Nevertheless, the gradual increase in the size of the lineage can be clearly inferred. The lineage then further expanded to seven clearly defined FT clades within the Poales (but not the other commellinid crown groups such as the Zingiberales), and then to twelve conserved clades within the Poaceae. Interestingly, FT1, FT2 and FT10 remained unduplicated during the evolution of the Poales and Poaceae, suggesting strong purifying selection to maintain them as single copy genes, while the FTX and FTY lineages acted as a ‘sandpit’ for sub- or neo-functionalization in grass genomes. The expansion to five FT lineages in most monocots implies a radical change in the way that FT proteins were used to regulate development, as does the subsequent increase in the Poales/Poaceae. An interesting question is whether this represented a partitioning of the prototypical FT function between multiple proteins (i.e. sub-functionalization) or whether FT proteins acquired new functions (i.e. neo-functionalization).

One way in which function and neo-function can be inferred is through analysis of protein motifs and domains. Our analysis identified key conserved residues that define each the MFT, TCB and FT proteins (Fig. 6, Supplementary Figure 3), which we used to assess possible sub- and neo-functionalization in monocot FT lineages. Our analysis strongly suggests that FT1 and FT2 maintain the original functionality of FT in angiosperms; these proteins show only minor changes in protein sequence relative to ‘EuFT’ proteins. However, this original function has presumably been sub-divided between FT1 and FT2, consistent with the analysis of FT1 and FT2 in model grasses [30, 33, 49–51]. Conversely, the FT10, FTX and FTY lineages show clear evidence of neo-functionalization, with dramatic changes to these conserved residues. The FT10 and FTX proteins appear to have modified 14-3-3 binding motifs, but are conserved in the P-loop motif, suggesting that they may still function as FT-like activators. These FTs may not act via 14-3-3-dependent complexes, but potentially via other proteins known to interact with FTs such as the TCP transcription factors [52–54]. Conversely, FTY proteins have better conservation in the 14-3-3 binding motifs, but show greater divergence in the P-loop region particularly in the key amino acids 134 and 138, which suggests that they may act as TCB-style repressors, rather than activators of development.

In terms of the expansion of the FTX and FTY lineages in the Poaceae, the evidence is mostly suggestive of sub-functionalization, with no major protein sequence differences between paralogs, with the exception of FT7/8, which appears to have completely lost the 14-3-3 binding motifs. Given the known diverse roles of the EuPEBPs, which act broadly as developmental checkpoints for the integration of environmental signals, the expansion of the FT lineage suggests that individual genes may have become specialized to integrate specific environmental cues. There is certainly evidence for this idea among the studies of different FT genes in grasses. In *Brachypodium distyachon*, FT9 promotes flowering in response to short-day length [30, 55]. In wheat and barley, FT3 is expressed in response to short-day lengths [28], and it has been identified in barley that it may only promote part of reproductive development, rather than flowering per se [56]. Barley FT4 represses flowering under long days, which is consistent with its amino acid sequence [29]. Further understanding regarding the sub- or neo-functionalization of EuPEBPs, and particularly FT proteins, will become increasingly possible as more whole and pan-genome sequences become available. Identifying regulatory regions which have been maintained between the monocot lineages would provide valuable insights into the potential regulatory proteins and environmental or developmental drivers controlling the expression of different FT paralogues. Complementing this information with expression datasets under multiple environmental conditions will be a powerful tool to understand the variable roles of these FT genes. While the most FT paralogs in grasses remain uncharacterized, these data suggest that there is significant potential to improve yield in key crop species by improving environmental adaptation through the properties of FT complexes.

## Conclusions

Our phylogenetic analysis refines previous models of EuPEBP evolution in early land plants, demonstrating the algal origin of the family, and pin-pointing the origin of the FT/TFL1 clade at the base of monilophytes. We have further demonstrated how a core set of genes (MFT1, MFT2, FT and TCB) at the base of flowering plants has undergone differential evolution in the major angiosperm lineages. At its most dramatic, this differential evolution includes the radical expansion of the FT family in monocots into 5 core lineages, further re-duplicated in the grass family to 12 conserved clades. Our analysis shows that many grass FT proteins are strongly divergent from other FTs and are likely neo-functional regulators of development, and provides a platform for the functional characterization of this fascinating family of regulatory proteins in the key crop species wheat, rice and maize.

## Materials and methods

### Bioinformatic retrieval of EuPEBP sequences

EuPEBP family members were identified by BLAST searches against complete genomes from Phytozome (https://phytozome.jgi.doe.gov). BLAST searches were performed using the FT, TCB and MFT coding sequences from *Arabidopsis thaliana*. In the majority of cases, searching with any of these sequences returned the same top hits in each species, including all bona fide FT, TCB and MFT, with very low *E* values (1 × 10^− 50^ or below); some transcriptomes however returned no hits. Reciprocal BLASTs were performed with recovered sequences to confirm that only true homologues of the target gene had been identified.

Preliminary trees were assembled and used to guide the iterative interrogation of other genome and transcriptome databases, particularly those generated by the 1KP project (https://db.cngb.org/onekp). For transcriptome datasets, we BLASTed each major taxonomic group separately. Generally, only closely related sequences were identified through this approach, and no cut-off was used. For non-annotated sequences from transcriptome datasets, we searched translations across all 6 reading frames to identify ORFs, and the longest ORFs were extracted for alignment. All sequences are listed in Supplementary File 1.

### Alignment

Alignments were initially performed in BioEdit [57] using ClustalW [58] with default settings. Full-length nucleotide sequences were toggled to amino acid sequences for alignment, which were manually refined as necessary. Alignments were stored as nucleotide-level sequence, allowing us to generate coherent nucleotide and amino acid alignments from the same alignments. Complete or incomplete sequences from transcriptome databases were then added, using the scaffold of the full-length sequences to align these sequences correctly. In order to identify the optimal phylogenetic reconstruction for each gene family, we created sub-sets of the full alignments at both nucleotide and amino acid levels. The resultant alignments are provided in Additional Files 3, 5, 6, 7. Pairwise protein identities were calculated using BioEdit. with the ‘Protein identity matrix’ function.

### Phylogenetic analysis

For each alignment, we trimmed the alignments to remove poorly conserved regions and then performed preliminary phylogenetic analyses to explore the topology of the tree and the effect of inclusion or exclusion of various groups of sequences. We found that using amino acid alignments generated extensive polytomies, so we restricted ourselves to nucleotide-level analyses. Once we were satisfied with the initial alignment, we used jModelTest 2.1 [59] to identify the most likely model of evolution for the alignment in question, as judged by the Akaike information criteria, Bayesian information criteria and decision theory, or a majority of these where there was not complete agreement. We then implemented maximum likelihood analysis in PhyML [60] using the optimal model. Where trees contained obvious mistakes, we amended the alignments, re-ran jModelTest, and repeated the analyses in PhyML. When a final alignment was arrived at, we then ran maximum likelihood analyses using the optimal model, with 250 bootstraps. Trees were visualized and modified using FigTree 1.4.2.

## Supplementary Information

**Additional file 1:.**

**Additional file 2:.**

**Additional file 3:.**

**Additional file 4:.**

**Additional file 5:.**

**Additional file 6:.**

**Additional file 7:.**

**Additional file 8:.**

## Data Availability

All data generated or analysed during this study are included in this published article and its additional files, or will be made available upon request.
