# Supplementary material for: Asymmetric expansions of FT and TFL1 lineages characterize differential evolution of the EuPEBP family in the major angiosperm lineages
Source: BMC Biol. 2021 Aug 31;19:181. doi: 10.1186/s12915-021-01128-8 (PMC8408984; doi:10.1186/s12915-021-01128-8)
Supplement: Supplementary file 2 — Additional file 2:. [file 12915_2021_1128_MOESM2_ESM.docx]

10 20 30 40 50 60

....|....|....|....|....|....|....|....|....|....|....|....|

AtFT SINIRDPLIVSRVVGDVLDPFNRSITLKVTYGQREVTNGLDLRPSQVQNKPRVEIGGEDR

AtTFL1 GTRVIEPLIMGRVVGDVLDFFTPTTKMNVSYNKKQVSNGHELFPSSVSSKPRVEIHGGDR

AtMFT MAASVDPLVVGRVIGDVLDMFIPTANMSVYFGPKHITNGCEIKPSTAVNPPKVNISGHSD

CiMFT SSTSAAALASGNIIGDVVDTFEPSLELKVSYGHKLVSDGVELTPAESSSVPAVEIGGKAG

MkMFT MSKPNEALVLGRVIGDVIDSVRPPADLKVQYDSRKVTNGAQLKPSETAAAPVVEIAGMVS

MeMFT HRTAMEQLKQAKIIGDVIDPFVQSVDMKVLYGSREVANEVELTPTQAASHPVVEIHGKKG

McMFT TDDSSPSLLETTIIGDIIDAVQKQLSLLVKYGENKVKNGIRMMPEDTTEVPQVTIGSSEE

ZsMFT MAKYSEALQVGRVIGDVLDPFTPSVDLKVEYNGRRVNNGVELKPSETASTPKVEIAGRAG

KnMFT VSISRFDATAVSLIADMLDAFTPSVALSIAYEGMPIQSGAELSPSEAEVFPTVRVGGSEN

CsPEBP1 AKEARKPTATSDYPDQLSDTTVLAAAKVIPDVVSRVTDTATLTIEYDGKPEEPTITIAGT

CsPEBP2 ATVAKFLEVNAGVQNAAALPTLVTYANHDVVPGEHFQSSRDAFVLNPPPAVSWSAIRPPL

DsPEBP QQPAGVPQHVVSGINDVVPFLEGTWELVIKYGGEVISQGEHIPVDTPALSKPPQLDIRPH

70 80 90

....|....|....|....|....|....|

AtFT NFYTLVMVDPDVPSPSNPHLREYLHWLVTDIPATTGTTFGNEIVCYENPSPTAGIHRVVF

AtTFL1 SFFTLVMIDPDVPGPSDPFLKEHLHWIVTNIPGTTDATFGKEVVSYELPRPSIGIHRFVF

AtMFT ELYTLVMTDPDAPSPSEPNMREWVHWIVVDIPGGTNPSRGKEILPYMEPRPPVGIHRYIL

CiMFT DLYTLVMTDPDAPDPKNPTKREWLHWIVKDIPGGTGAAAGKEVVAYMGPSPPSGVHRYAF

MkMFT ELYTLVMVDPDAPSPSEPLFREWLHWIVVDIPGSSVPPAGKEVVEYNGPTPPIGIHRYVF

MeMFT MLYTLLMVDPDAPSPDAPAFREWLHWMVVDIPGIPAENTVKEVVQYNGPTPPLGLHRYVL

McMFT PLYTLVMVDPDVPSPNEPSAREWLHWIVVDIPGTSVPGTGTEVVEYNNPLPPVGIHRYVF

ZsMFT ELYTLVMVDPDAPSPSEPSAREWLHWIVTDIPGGTVPESGTVVVDYNGPTPPIGIHRYVF

KnMFT DLYTLVLVDPDAPSPEAPSEAEWLHWLVTNIPGGQEATAGTDVVPYSGPTPPVGRHRYVF

CsPEBP1 DTYTLVMVDPDAPSPDHPKYRFFLHWLVVNIPGVDVNRG~EVVTAYMGPSPPKGTHRYVF

CsPEBP2 DPTKHRMFDPDAPEPAPGDGATPGANAVWTTHGGNAATAPRLAVPYEPPVPPKGTHRYVF

DsPEBP DLAQGRMIDPDAPDPENPSAKEWLHWYRVNISGLELLRIAEDVVEYSPPHPVSGTHRYIF

130 140 150 160 170

....|....|....|....|....|....|....|....|....|....|.

AtFT ILFRQLGRQTVYAPGWRQNFNTREFAEIYNLGLPVAAVFYNCQRESGCGGR

AtTFL1 VLFRQKQRRVIFPIPSRDHFNTRKFAVEYDLGLPVAAVFFNAQRETAARKR

AtMFT VLFRQNSPVGLMVPPSRANFSTRMFAGHFDLGLPVATVYFNAQKEPASRRR

CiMFT IVFKQAAPLDMEMPRSRANFNTRNFAKDHLLGSSVAAVYFTAQP#

MkMFT VLFKQKGPITVTPPSTRKSFNTRAFAQQYDLGAPVAAVYFNAQKEGPGGRR

MeMFT VLFAQEEAISRTVPAVRKSFHTREFAKEMKLGLPVAALFFLAQPEGKR

McMFT VLFKQPGPLKVEPPKKRNNFSTRLFAKEHCLGTPVAVT#

ZsMFT VIFKQKGPLKVTPPESRKNFNTRWFAVTYDLGLPVSAVYFNAQKEGGSAT

KnMFT LLYEQAYQIEAQPPTQRNRFKAQMFAQEHELGDPVAASLFYASAAKAERET

CsPEBP1 LLYKQNGRVSAKNPHSRQNFTLHQFTKEHSLGDPAAAVFFYSAPEE

CsPEBP2 VLFEQAGEKPIVVSEGAETQRREWDLKAFLAKNPGARAAAVNHLTCSPPSK

DsPEBP LLYAQEHEHQRPSLLRMHLTDHAVEGPVTIGLHRMCFDHRAWATRHDLHGP

**Additional File 1**

Alignment of charopyte and chlorophyte EuPEBP sequences with MFT, TFL1 and FT from *Arabidopsis* *thaliana* (At). Ci = *Coleochaete* *irregularis*, Mk, Me, Mc = *Mesotaenium* *kramstei* / *endlicherianum* / *caldariorum*, Zs = *Zygnema* sp., Cs = *Coccomyxa* *subellipsoidea*, Ds = *Dunaliella* *salina*. # = missing sequence. Alignment was trimmed to conserved regions used elsewhere in this study.
