# Supplementary material for: Asymmetric expansions of FT and TFL1 lineages characterize differential evolution of the EuPEBP family in the major angiosperm lineages
Source: BMC Biol. 2021 Aug 31;19:181. doi: 10.1186/s12915-021-01128-8 (PMC8408984; doi:10.1186/s12915-021-01128-8)
Supplement: Supplementary file 4 — Additional file 4:. [file 12915_2021_1128_MOESM4_ESM.docx]

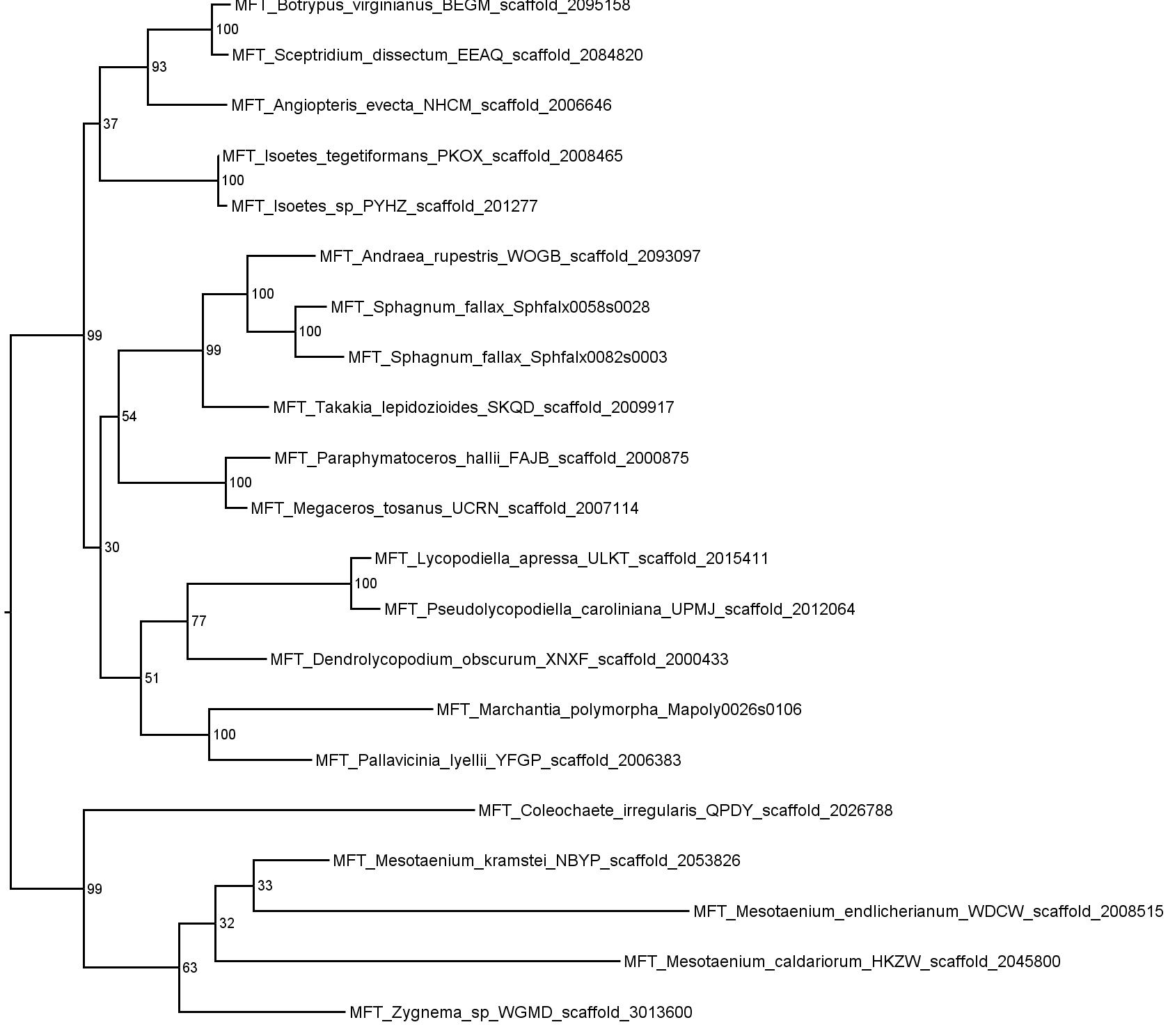


**Additional File 4: Basal land plant MFT phylogeny**

Nucleotide-level maximum likelihood analysis implemented in PhyML using a TIM2ef+G+I model, on the basal land plant MFT sequences (21 sequences, 519 characters). The tree was rooted with charophyte algal sequences. Phylogram showing the most likely tree, including bootstrap values at all nodes.
