# Supplementary material for: Asymmetric expansions of FT and TFL1 lineages characterize differential evolution of the EuPEBP family in the major angiosperm lineages
Source: BMC Biol. 2021 Aug 31;19:181. doi: 10.1186/s12915-021-01128-8 (PMC8408984; doi:10.1186/s12915-021-01128-8)
Supplement: Supplementary file 8 — Additional file 8:. [file 12915_2021_1128_MOESM8_ESM.docx]

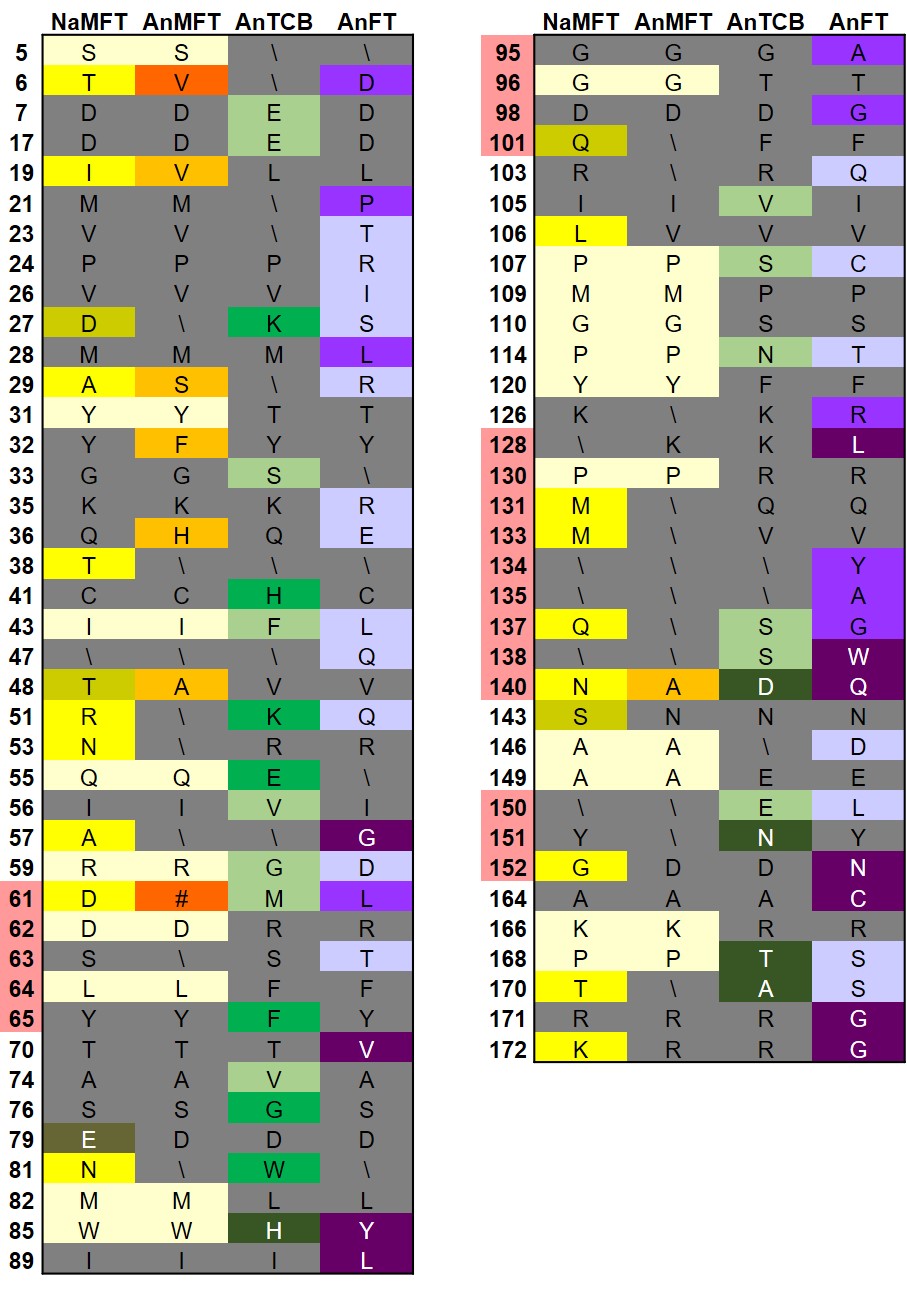


**Additional File 8: Unique characteristics of EuPEBP protein families**

Diagram showing residues at which major EuPEBP families have unique amino acids, not found in other families. NaMFT = non-angiosperm MFT. AnMFT = angiosperm MFT, AnTCB = angiosperm TCB and AnFT = angiosperm FT (without monocot proteins). Unique residues are coloured, non-unique (or non-conserved) residues are shown in grey. The lighter shades are consensus amino acids (50%>75%), the mid shades are conserved (75%>90%) and the dark shades are highly conserved (>90%). Amino acids that are shared between all MFT proteins are shown in beige. The position of the 14-3-3 interaction, P-loop and LYN triad motifs are shown in pink shading of the number column. # = residue absent, \ = no consensus.
